# SARS-CoV-2 nanobodies 2.0

**DOI:** 10.1038/s41392-021-00632-1

**Published:** 2021-05-22

**Authors:** Fabien Labroussaa, Joerg Jores

**Affiliations:** Institute of Veterinary Bacteriology, Department of Infectious Diseases and Pathobiology, University of Bern, Bern, Switzerland

**Keywords:** Structural biology, Infectious diseases

In a recent study published in Science, Koenig et al.^1^ reported on rationally engineered biparatopic nanobodies targeting the receptor-binding domain (RBD) of the spike protein that not only efficiently neutralize SARS-CoV-2, but additionally suppress mutational escape.

Population growth, increased travel and climate change foster epidemic and pandemic threats by (re)emerging viruses. Although black swan events cannot be predicted, we should be prepared to use the latest advancements in science to combat emerging viruses. Within the past decade, we have seen several viral outbreaks including the latest SARS-CoV-2 pandemic. Safe effective and scalable vaccines represent undisputedly the most cost-effective control measure. But it is not always possible to develop and roll out a vaccine, and certain medical or age-related conditions could exclude people from getting their jabs. Passive immunizations by antibody-related molecules complement vaccines, especially in high-risk persons.

**Fig. 1:**
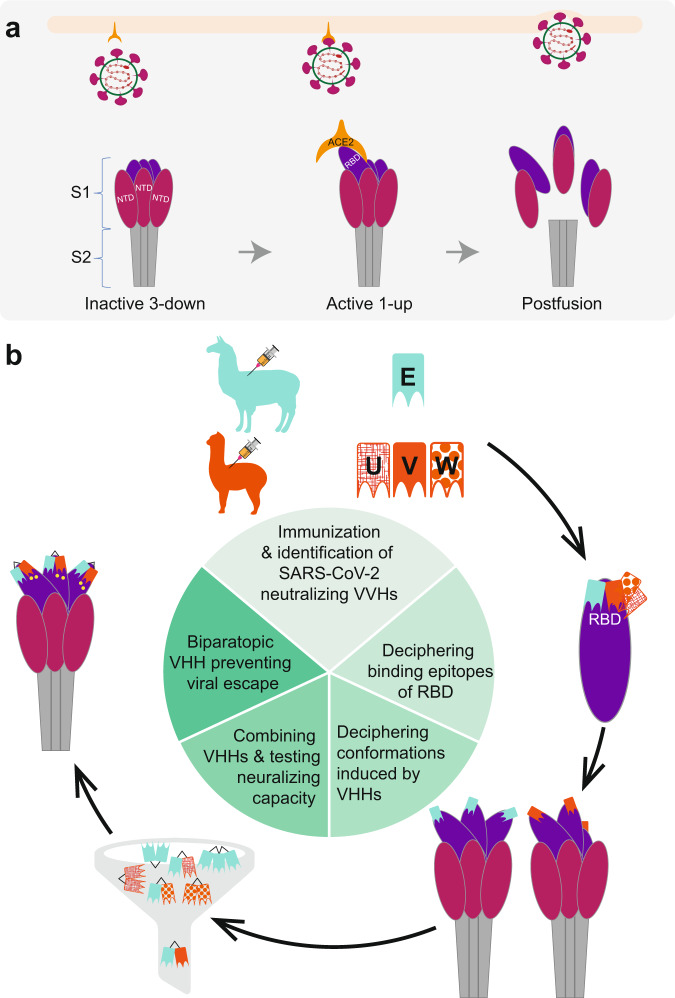
Cartoon showing the rational engineering of nanobodies. **a** The spike protein consisting of the S1 and S2 subunits. The N-terminal domain (NTD) and the receptor-binding domain (RBD) of the S1 unit during the process of binding to the angiotensin-converting enzyme 2 (ACE2) receptor are displayed (left to right). The ACE2 receptor can only bind to RBD in the up position, the binding of ACE2 triggers the fusion machinery including dissociation of the subunits; **b** workflow to generate SARS-CoV-2-neutralizing biparatopic antibodies that prevent viral escape

Single heavy chain type antibodies have been described for camelids and their variable antigen-specific binding region is termed VHH domain or nanobody. This binding region contains three enlarged complementarity-determining regions (CDRs), providing an expanded antigen-interacting surface, which has a rather convex paratope making it well-suited for binding epitopes that are inaccessible for IgG. Nanobodies are easy to manufacture using prokaryotic expression systems, have an enhanced thermal- and conformational stability, efficiently refold, retain their binding characteristics and induce only a low response from the human immune system, which makes them attractive for biomedical applications.^2^ Koenig et al. immunized one alpaca and one llama with the RBD of SARS-CoV-2 as well as formalin-inactivated SARS-CoV-2 to generate SARS-CoV-2-neutralizing single-chain antibodies. Most neutralizing antibodies against SARS-CoV-2 target the trimeric spike (S) protein catalyzing the fusion between host and viral membranes.^3^ The S protein consists of the subunits S2, anchored in the virus envelope, and S1 comprising the N-terminal domain (NTD) and the RBD harboring the receptor-binding motif (RBM) that interacts with the dimeric ACE2^4^ (Fig. 1a). Immunization of different animal species allowed tapping into different antibody repertoires, increasing the likelihood to fish out different binders. They identified 10 RBD-specific antibodies by enzyme-linked immunosorbent assay, including four nanobodies (VHHs E, U, V, and W) with effective neutralizing capacity as shown by in vitro assays employing SARS-CoV-2 pseudotyped vesicular stomatitis virus. The llama-derived nanobody VHH E bound to a different epitope than the three alpaca-derived nanobodies VHHs U, V, and W, as shown by surface plasmon resonance-based binding competition assays (Fig. 1b). Subsequently, the detailed binding epitopes of the four VHHs with the RBD were revealed using X-ray crystallography. This way, the authors were able to pinpoint not only the different binding epitope residues, but also to predict their competing epitopes with the ACE2 receptor and compare the four nanobodies to other already published neutralizing binders. Flow cytometry-based competition assays employing ACE2 expressing HEK 293T cells confirmed the predicted competition ability of the nanobodies. The involvement of VHHs’ individual CDRs towards their binding epitopes within the RBD was mapped and it was shown that glycosylation of RBD’s amino acids impacted binding.

The trimeric spike S2 subunit can exist in different conformational states; all RBD down (three-down), individual RBDs up, and all RBDs up (three-up)^5^, the latter is the least populated conformation. Binding to ACE2 requires at least one up conformation and triggers the fusion events (Fig. 1a). In order to decipher possible conformational changes induced by the nanobodies VHH E and V, cryo-electron microscopy was used in combination with a stabilized engineered spike to monitor conformational consequences resulting from binding. Interestingly, VHH E bound to all RBDs in the three-up conformation, whereas VHH V bound to the two-up conformation (Fig. 1b). Next, it was shown that the VHH E and V triggered the fusion machinery. Therefore, HEK 293 cells that can be induced to express the SARS-CoV-2 spike protein on the surface and labeled with green or red fluorescent proteins were mixed with and without the nanobodies allowing visualization of cell fusion induction by the VHHs E, U, and V. In contrast, the fusion of fluorescent HEK 293 cells expressing ACE2 or SARS-CoV-2 spike was reduced to 50% in the presence of VHH E, confirming its neutralization capacity. As expected, the neutralizing capacity of combinations of nanobodies targeting different RBD epitopes (E-V, E-U, E-W) was enhanced, whereas the capacity of combinations targeting the same epitope was additive. Subsequently, multivalent nanobodies targeting different RBD epitopes linked by flexible linkers were produced and the biparatopic nanobodies resulted in much better binding and neutralization capacity. Interestingly, the inclusion of VHH E in the biparatopic nanobody VHH VE resulted in the occupation of all six binding sites in the three-up conformation (Fig. 1b). Multimerization of individual VHHs resulted in enhanced affinity and up to 100-fold neutralization activity. Afterward, the authors showed in a series of elegant experiments that the different nanobodies neutralized by different mechanisms including different affinities for ligands and/or secondary proteolytic processing by proteases. Finally, they tested mutual escape, an important test given the current emergence of mutants and their possible implication in vaccine efficacy. They clearly showed that the biparatopic VHHs EV and VE were superior to VVH E+U, E+V as well as E+W in preventing mutual escape, rounding up this nice piece of work (Fig. 1b). As in vitro data do not necessarily translate into in vivo data, the engineered antibodies await their functional testing using in vivo models. Although several nanobodies have been reported mainly based on synthetic libraries, the stability of nanobodies and the ease of production gives the nanobodies developed a good chance to turn into a viable commercial product to combat SARS-CoV-2 infections. In summary, Koenig et al. performed an impressive study, reporting on the rational engineering of nanobodies on the basis of well-characterized binding properties and architecture in combination with insight into the mechanistic mode of action related to spike’s ability to trigger fusion. This paper provides a good example of translating basic research into the development of therapeutic binders for a pandemic pathogen.
